# Suppressing adipocyte inflammation promotes insulin resistance in mice

**DOI:** 10.1016/j.molmet.2020.101010

**Published:** 2020-05-11

**Authors:** Qingzhang Zhu, Yu A. An, Min Kim, Zhuzhen Zhang, Shangang Zhao, Yi Zhu, Ingrid Wernstedt Asterholm, Christine M. Kusminski, Philipp E. Scherer

**Affiliations:** 1 Touchstone Diabetes Center, Department of Internal Medicine, University of Texas Southwestern Medical Center, Dallas, TX, USA; 2 Department of Biological Sciences, School of Life Sciences, Ulsan National Institute of Science and Technology, Ulsan, South Korea; 3 Department of Physiology/Metabolic Physiology, Institute of Neuroscience and Physiology, Sahlgrenska Academy at University of Gothenburg, Gothenburg, Sweden

**Keywords:** Adipocyte, Inflammation, Insulin resistance

## Abstract

**Objective:**

Obesity-induced insulin resistance is closely associated with chronic subclinical inflammation in white adipose tissue. However, the mechanistic involvement of adipocyte-derived inflammation under these disease conditions remains unclear. Our aim was to investigate the relative inflammation-related contributions of adipocytes and macrophages to insulin sensitivity.

**Methods:**

RIDα/β is an adenoviral protein complex that inhibits several inflammatory pathways, including TLR4, TNFα, and IL1β signaling. We generated novel mouse models with adipocyte-specific and macrophage-specific doxycycline (dox)-inducible RIDα/β-transgenic mice (RID^ad^ and RID^mac^ mice, respectively).

**Results:**

RIDα/β induction significantly reduced LPS-stimulated inflammatory markers, such as *Tnf*, *Il1b*, and *Saa3* in adipose tissues. Surprisingly, RID^ad^ mice had elevated levels of postprandial glucose and insulin and exhibited glucose intolerance and insulin resistance, even under chow-fed conditions. Moreover, the RID^ad^ mice displayed further insulin resistance under obesogenic (high-fat diet, HFD) conditions despite reduced weight gain. In addition, under pre-existing obese and inflamed conditions on an HFD, subsequent induction of RIDα/β in RID^ad^ mice reduced body weight gain, further exacerbating glucose tolerance, enhancing insulin resistance and fatty liver, and reducing adiponectin levels. This occurred despite effective suppression of the inflammatory pathways (including TNFα and IL1β). In contrast, RID^mac^ mice, upon HFD feeding, displayed similar weight gain, comparable adiponectin levels, and insulin sensitivity, suggesting that the inflammatory properties of macrophages did not exert a negative impact on metabolic readouts. RIDα/β expression and the ensuing suppression of inflammation in adipocytes enhanced adipose tissue fibrosis and reduced vascularization.

**Conclusion:**

Our novel findings further corroborate our previous observations suggesting that suppressing adipocyte inflammation impairs adipose tissue function and promotes insulin resistance, despite beneficial effects on weight gain.

## Highlights

• Suppression of adipocyte inflammation promotes insulin resistance under both normal and obesogenic conditions.
• Suppression of adipocyte inflammation with pre-existing obesity further aggravates insulin resistance.
• Suppression of macrophage inflammation has little effect on obesity-induced insulin resistance.
• Adipocyte inflammation may therefore be required to maintain normal adipose tissue function.

## 1 Introduction

Obesity-associated insulin resistance is a major risk factor for multiple comorbidities, including type 2 diabetes, cardiovascular diseases, and several types of cancers [1,2]. Low-grade and sustained inflammation is frequently seen in obese white adipose tissue (WAT). Insulin resistance and adipose tissue inflammation correlate very well under many conditions, but a causal relationship between the two remains to be shown [3,4]. Conventional treatments for obesity and diabetes, including metformin, GLP1 receptor agonists, and TZDs, lead to improvements in insulin sensitivity while also providing anti-inflammatory benefits. In contrast, the effects of anti-inflammatory therapies on insulin sensitivity have not been observed other than those reported for high doses of salicylate [[5], [6], [7], [8]]. Interventions in clinical trials, such as anti-TNFα antibodies, statins, glucocorticoids, and IL1β receptor antagonists, not only fail to improve but frequently even worsen insulin sensitivity. They may however lower hyperglycemia independent of insulin sensitivity [[9], [10], [11], [12], [13]]. In this regard, it is important to better understand the basic mechanisms tying adipocyte inflammation to insulin resistance.

Inflammation is a complex immune response orchestrated by many cell types in the microenvironment of target organs. Adipose tissue displays widespread heterogeneity at the cellular level, and this heterogeneity is further regulated under metabolically challenging conditions. In fat depots, approximately 20–40% of all cells consist of adipocytes, while the rest are composed of preadipocytes, endothelial cells, and many immune cells taking part in both the innate and adaptive immune systems [14]. Among these cells, macrophages are a very prominent immune cell type in adipose tissue. While they represent less than 10% of all cells in the lean state, they can reach up to 50% of all cells in the obese state [15,16]. In addition, obesity promotes a transition of macrophages from an M2-like cell type to an M1-like population. M1 macrophages release factors, such as TNFα and IL1β, that promote inflammation and are thought to potentially contribute to insulin resistance; in contrast, M2 macrophages are activated by Th2 cytokines, resolve inflammation, and induce extracellular matrix (ECM) remodeling [17]. In the context of obesity, adipocytes become hypertrophic and reduce their production and release of the insulin-sensitizing factor adiponectin. By acquiring macrophage-like properties, these hypertrophic adipocytes start producing many inflammatory cytokines and chemokines, conventionally thought to be released by macrophages, including TNFα, IL1β, and MCP1. To further synergize the immune response, these factors recruit circulating leukocytes and activate proinflammatory M1 macrophages in WAT.

However, it is difficult to dissect the individual contributions of adipocytes or macrophages to inflammation-related insulin resistance in obesity. A genetically induced model of insulin resistance in adipocytes promotes the accumulation of proinflammatory macrophages via MCP1, suggesting that insulin resistance in adipocytes could be the cause of adipose tissue inflammation [18]. Our previous study showed that a minimal level of inflammation within the adipocytes is essential for proper adipose tissue development. Constitutively blocking inflammation causes adipose tissue dysfunction and thus impairs glucose metabolism [19].

RIDα/β (receptor internalization and degradation) is a transmembrane heterotrimeric complex of 10.4kD/14.5kD subunits encoded by the adenovirus E3 region that strongly inhibits inflammatory pathways including the TLR-4, TNFα, and IL1β pathways [20]. In this study, we used RIDα/β to modulate inflammation specifically in adipocytes or macrophages in an inducible fashion under different conditions and demonstrated that adipocyte inflammation, rather than macrophage inflammation, is critical to maintain normal adipose tissue function. Thus, suppressing adipocyte inflammation promotes insulin resistance under both normal and obese conditions.

## 2 Materials and methods

### 2.1 Mice

Mice were housed at 22 °C with 12-h light–dark cycles and free access to water and food (chow #5058, Lab Diet; 60% high-fat diet [HFD] paste), doxycycline (dox, 600 mg/kg), and HFD (BioServ). All of the mice had a C57BL/6J background. All of the mouse protocols were approved by the Institutional Animal Care and Use Committee of the University of Texas Southwestern Medical Center (APN: 2015-101207G). Adipoq-rtTA [21], Csf1r-rtTA, and TRE-RIDα/β mouse lines were generated in house. The Csf1r-rtTA mice were generated by expressing rtTA (the reverse tetracycline-controlled transactivator) under the control of 8.0-kb mouse Csf1r (colony-stimulating factor 1 receptor) promoter, which was injected into fertilized (C57BL/6N) F1 mouse eggs at the UT Southwestern Medical Center transgenic core. The resulting transgenic mice were bred to C57BL/6J mice. Founders were screened for expression and specificity by crossing mice to TRE-GFP reporter mice. For the TRE-RIDα/β mice, the 2A peptide derived from porcine teschovirus-1 (P2A) was used to splice together the α and β subunits. The RIDα-P2A-RIDβ construct was subcloned into the TRE vector [21,22] with a rabbit β-globin 3′-UTR. The construct was injected into fertilized (C57BL/6N) F1 mouse eggs at the UT Southwestern Medical Center transgenic core. The resulting transgenic mice were bred to C57BL/6J mice. Founders were screened for expression and specificity by crossing mice to Adipoq-rtTA mice and ensuring specific and inducible expression in the adipocytes. Male mice were used. The experiments were started with the mice at eight weeks of age. For obesogenic conditions, the mice were fed HFD-dox for 10 or 26 weeks as indicated. For obese preconditioning, the mice were fed HFD (lacking dox) for 30 days, then switched to HFD-dox for another 10 weeks. For LPS treatment, the mice received LPS at a dose of 0.3 mg/kg body weight (i.p.) for 6 h and were then sacrificed and the tissues were collected for further analysis. Serum VEGF was measured with a kit from Millipore (MCYTOMAG-70K), and serum insulin was determined with a kit from ALPCO (#80-INSMSU-E10) following the manufacturers' instructions.

### 2.2 Oral glucose tolerance test (OGTT), insulin tolerance test (ITT), and arginine tolerance test (ATT)

OGTT and ITT tests were conducted following standard protocols [23]. Blood glucose levels were monitored at the indicated time points. ATTs were conducted with dual arginine injections administered at time 0 and 10 min [24]. The first stimulus emptied the releasable pool of granules and the second stimulus challenged the ability to replenish the pool. Blood was collected from the tail vein at the indicated time points for later analysis.

### 2.3 Histology and immunostaining

Mouse tissues were dissected and fixed in 10% formalin overnight. Paraffin processing, embedding, sectioning, hematoxylin/eosin staining, and trichrome staining were performed by John Sheldon at UTSW Medical Center. Immunostaining was performed following standard protocols, with the following antibody against endomucin (sc-65495, RRID: AB_2100037). Three to four biological replicates for histological analyses are shown. Images were acquired with an Olympus FSX100 microscope and analyzed by ImageJ software.

### 2.4 Bone marrow-derived macrophage (BMDM) isolation, differentiation, and treatment

BMDM from WT and RID^mac^ mice were isolated and differentiated according to a standard protocol previously described [25]. After differentiation, RIDα/β was induced with dox (1 μg/ml) overnight, and then the cells were treated with LPS (100 ng/ml) or IL4 (10 ng/ml) for 24 h. After treatment, the cells were collected for downstream analysis.

### 2.5 Western blotting analysis

Serum samples (0.5 μl per lane) were boiled at 95 °C in a protein-loading buffer and applied to 4–12% sodium dodecyl sulfate-polyacrylamide gel (SDS-PAGE). Antibodies against mouse adiponectin (homemade) following fluorescent-conjugated secondary antibodies (IRDye, LI-COR, Lincoln, NE, USA) were used to determine the adiponectin levels. Images were acquired and the fluorescent density was quantified utilizing a LI-COR Odyssey Imager (LI-COR, Lincoln, NE, USA).

### 2.6 RNA isolation and quantitative RT-PCR (qPCR)

Total RNA was extracted using the traditional TRIzol method. Quantitative gene expression was conducted by two-step quantitative RT-PCR using iScript cDNA synthesis kits (#170–8891, Bio-Rad) and SYBR Green PCR Master Mix (Applied Biosystems). The mRNA expression levels were determined using the ΔΔCT method and normalized to the housekeeping genes *Rps16* and *Rps18*. Primers were from the Harvard PrimerBank (https://pga.mgh.harvard.edu/primerbank/).

### 2.7 Statistics

All of the data were expressed as mean ± SEM (∗∗∗∗p ≤ 0.0001, ∗∗∗p ≤ 0.001, ∗∗p ≤ 0.01, and ∗p ≤ 0.05). For two independent data sets, two-tailed Student's t-test was used. For multiple comparisons, one-way or two-way ANOVA were used with Bonferroni's multiple comparisons test. The statistical analyses were conducted using GraphPad Prism 8.0 (GraphPad Software, Inc., La Jolla, CA, USA).

## 3 Results

### 3.1 Suppressing adipocyte inflammation in adult mice results in insulin resistance under chow-fed conditions

**Figure 1:**
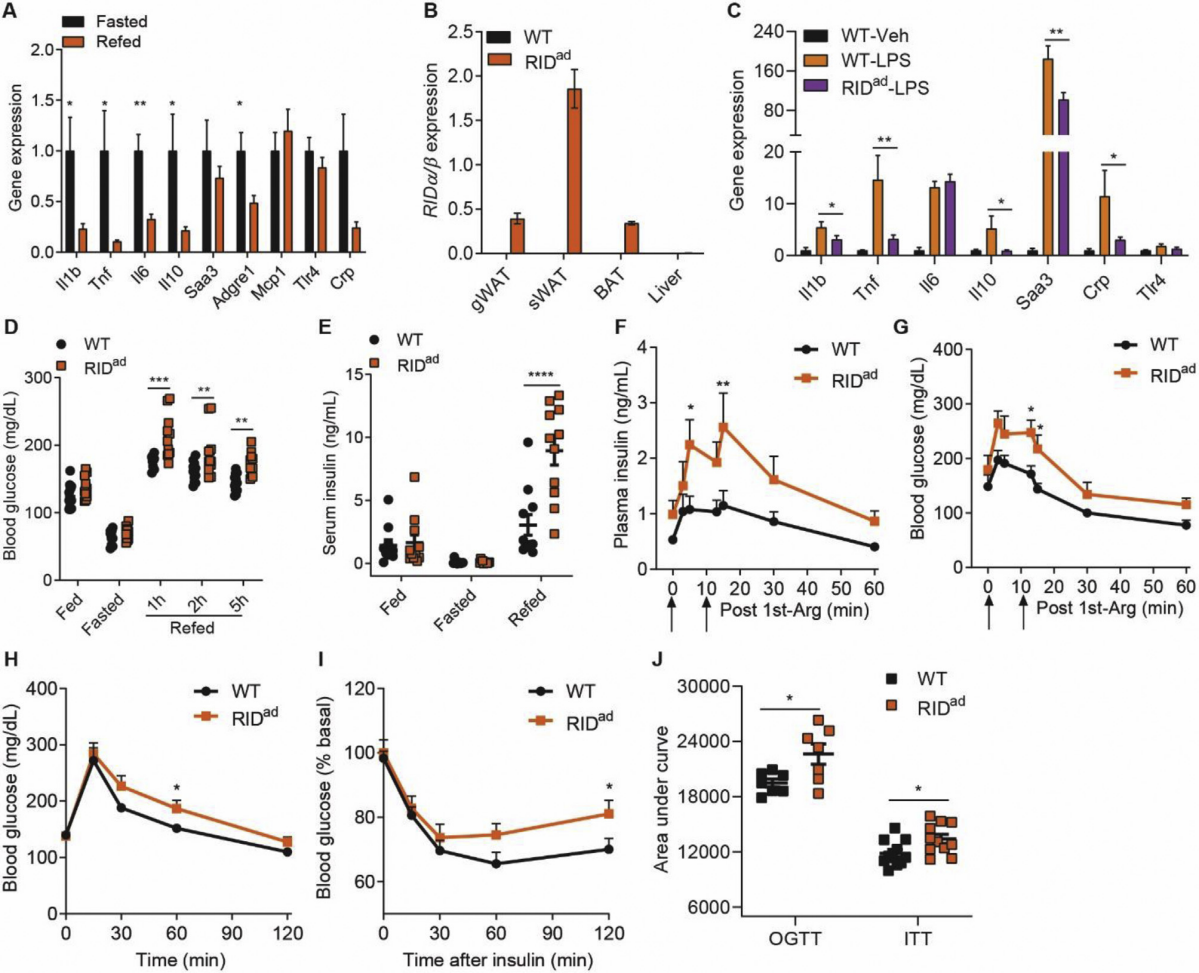
Suppressing adipocyte inflammation leads to insulin resistance under normal chow-fed conditions. (**A**) Suppression of inflammatory markers in gonadal WAT (gWAT) 2 h post-refeeding after overnight fasting. N = 8–10/group. (**B**) Adipocyte-specific expression of *RIDα/β* in mice after 2 weeks of dox induction. N = 10–14/group. (**C**) RIDα/β expression effectively lowers LPS-stimulated induction of inflammatory markers in gWAT of two-week dox-induced mice. N = 6/group. (**D-E**) RID^ad^ mice display postprandial hyperglycemia and hyperinsulinemia (2h) during an overnight fasting/refeeding procedure. N = 11/group. (**F-G**) RID^ad^ mice display enhanced arginine (Arg)-induced insulin release but maintain higher glycemia. N = 5/group. (**H-J**) RID^ad^ mice display impaired glucose tolerance (N = 7/group) and insulin tolerance (N = 11/group).

Adipose tissue exhibits a substantial inflammatory potential that exerts profound effects locally and systemically via the secretion of many inflammatory factors. In fact, adipose tissue inflammation is subject to normal physiological variations over a significant range. Indeed, adipose tissue inflammation, as judged by an array of markers, increases in the fasted state and is downregulated in the fed state. The feeding-suppressed inflammatory cytokines involve a number of inflammatory markers, including *Il1b*, *Tnf*, *Il6*, *Il10*, and *Adgre1* (which encodes F4/80) (Figure 1A). We thus wondered whether modulating adipocyte inflammation would systemically control glucose homeostasis in a similar way it does *in vivo* during refeeding. To address this, we generated mice that allowed us to express the anti-inflammatory factor *RIDα/β* specifically in adipocytes in a doxycycline (dox)-inducible fashion (hereafter we refer to these mice as RID^ad^ mice) (Figure 1B). RIDα/β displays potent anti-inflammatory activity by inhibiting several proinflammatory pathways, including IL1β and TNFα. Upon induction of RIDα/β, bacterial lipopolysaccharide (LPS)-stimulated inflammatory factors, such as *Il1b*, *Tnf*, *Il10*, *Saa3*, and *Crp,* are significantly downregulated in RID^ad^-gonadal WAT (gWAT) (Figure 1C), demonstrating that adipocyte RIDα/β effectively suppresses local inflammation in adipose tissues. Surprisingly, despite the suppressed inflammatory response, postprandial glycemia is significantly elevated in the RID^ad^ mice (Figure 1D). Similarly, the serum insulin levels are elevated (Figure 1E). Thus, adipocyte inflammation per se indeed exerts an influence on glucose homeostasis, as the suppression of adipocyte inflammation causes postprandial insulin resistance. Moreover, the RID^ad^ mice, despite displaying higher insulin levels upon arginine stimulation (Figure 1F), maintain higher glycemia (Figure 1G). In line with these observations, glucose disposal is impaired and insulin sensitivity is decreased in the RID^ad^ mice (Figure 1H–J). Thus, the local suppression of adipocyte inflammation leads to systemic insulin resistance even under chow-fed conditions.

### 3.2 Suppressing adipocyte inflammation promotes insulin resistance under obesogenic conditions

**Figure 2:**
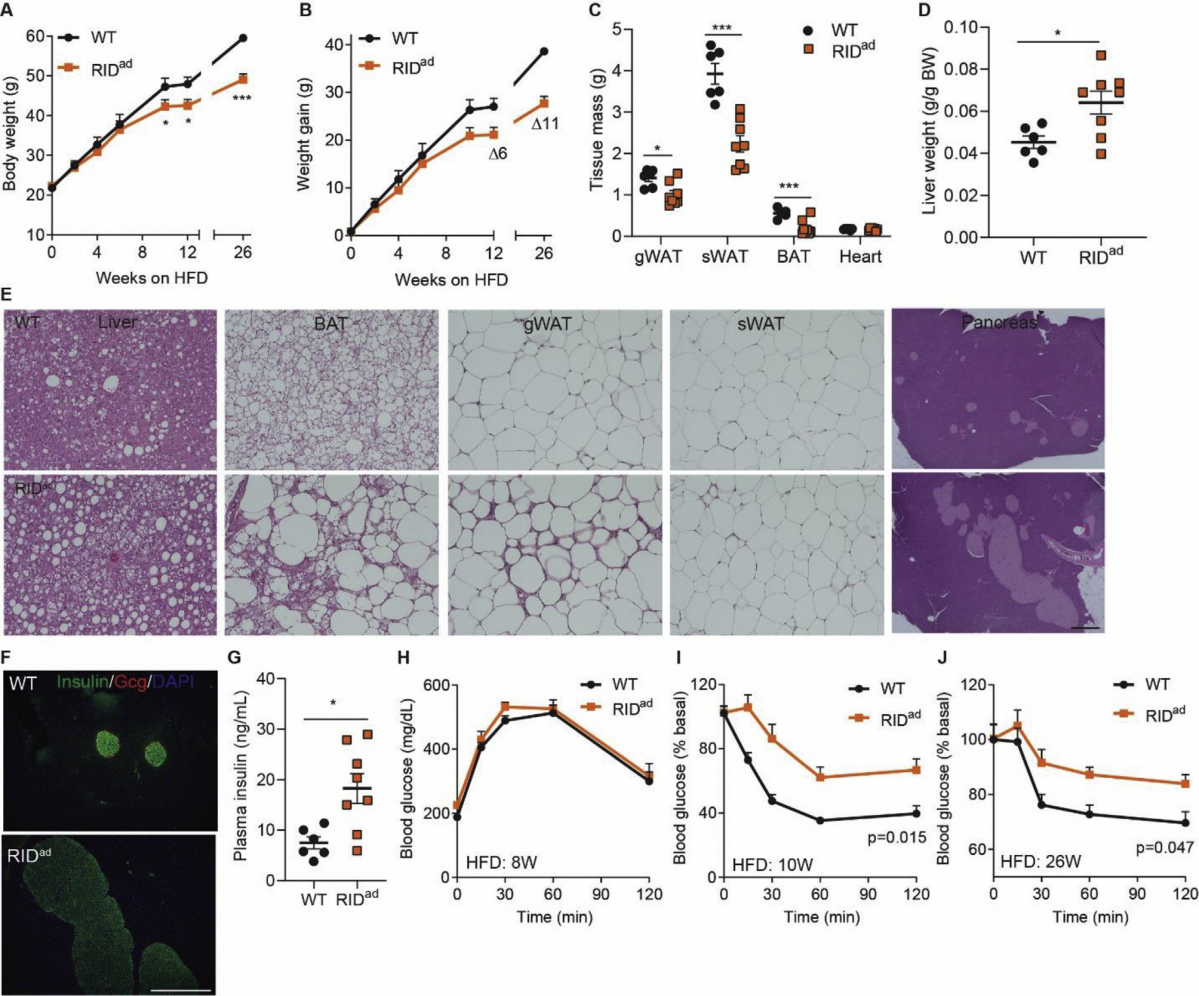
Suppressing adipocyte inflammation causes insulin resistance under obesogenic conditions. Mice were fed HFD-dox for 26 weeks. (**A-C**) RID^ad^ mice display less weight gain and reduced fat mass upon HFD-dox feeding. (**D**) RID^ad^ mice have elevated liver/body weight ratios. (**E**) H&E stain of liver, brown adipose tissue (BAT), gWAT, subcutaneous WAT (sWAT), and pancreas. Scale bar, 100 μm. (**F**) Insulin/glucagon (Gcg) double stain of the pancreas. Scale bar, 400 μm. (**G**) Elevated serum insulin levels in HFD-dox-fed RID^ad^ mice. (**H-J**) Similar glucose tolerance but impaired insulin tolerance in HFD-dox-fed RID^ad^ mice after 10 and 26 weeks of HFD-dox. N = 6–8/group for all of the statistical graphs.

Obesity is frequently associated with enhanced adipose tissue inflammation. Thus, the key question is whether suppressing inflammatory responses in adipocytes could be associated with beneficial effects. To test this, we fed the mice a dox-containing obesogenic high-fat diet (HFD-dox). As a result, we observe a lower body weight in the RID^ad^ mice that significantly diverges from control mice on an identical diet after eight weeks of HFD exposure (Figure 2A–B). This difference in body weight is associated with a reduction in fat mass (Figure 2C). Surprisingly, the RID^ad^ mice exhibit a higher liver/body weight ratio and hence a fatty liver. Moreover, RID^ad^ BAT (brown adipose tissue) turns markedly whiter and gWAT displays much greater macrophage infiltration, whereas subcutaneous WAT (sWAT) is less affected (Figure 2D,E). In addition, the pancreatic islets become hypertrophic with lower insulin content (Figure 2E,F) and serum insulin levels are elevated (Figure 2G). Although glucose tolerance is unaltered after 8 weeks of HFD-dox feeding in the RID^ad^ mice, these mice display much lower insulin sensitivity beyond 10 weeks of HFD-dox feeding all the way to the 26-week time point (Figure 2H,J). Thus, suppressing adipocyte inflammation promotes insulin resistance, despite being associated with reduced body weight under these obesogenic conditions.

### 3.3 Suppressing adipocyte inflammation causes further insulin resistance upon obese pre-conditioning

**Figure 3:**
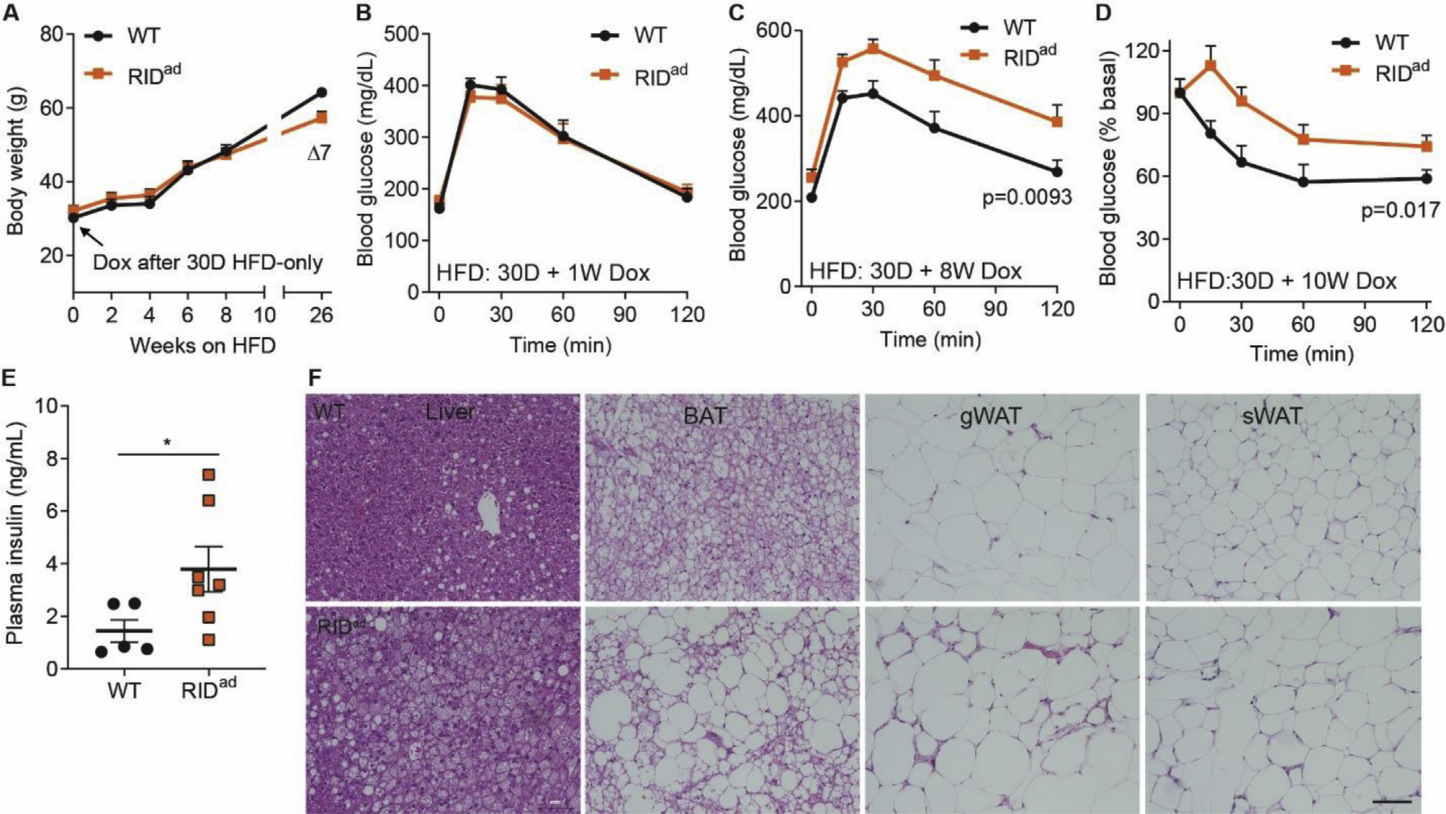
Suppressing adipocyte inflammation causes further insulin resistance upon obese pre-conditioning. (**A**) Mice were fed HFD only (no dox) for 30 days and then switched to HFD-dox for another 10 weeks. A slightly reduced body weight is observed in RID^ad^ mice. N = 9–12/group. (**B**) One-week acute induction of RIDα/β results in comparable glucose tolerance tests. N = 9–12/group. (**C-D**) Prolonged HFD-dox exposure (8 weeks) impairs glucose and insulin tolerance in RID^ad^ mice. N = 9–12/group. (**E**) Elevated serum insulin levels in RID^ad^ mice. N = 5–7/group. (**F**) H&E staining of liver, BAT, gWAT, and sWAT. Scale bar, 100 μm.

These observations are consistent with our previous findings obtained in aP2-RID mice [19], in which constitutive congenital inhibition of inflammation leads to insulin resistance under chow-fed and obesogenic conditions. We therefore wondered whether inflammation is required to maintain adipose tissue in the obese state or just for the initial stages of fat expansion. Thus, we initiated the suppression of inflammation at a point when adipose tissue inflammation is already pre-existing. Would we see beneficial effects in terms of glucose control? If so, would our anti-inflammatory regimen provide support to further explore pharmacological targets to suppress inflammation in adipose tissue with the hope of improving insulin sensitivity? We therefore pre-fed mice with HFD (lacking dox) for 30 days to initiate adipose tissue expansion toward obesity and adipose tissue inflammation. Thereafter, HFD-dox was applied. The RID^ad^ mice still gain less body weight in the subsequent weeks after the initiation of dox treatment (Figure 3A). However, the weight differential is reduced compared to the experiments when dox was present during the entire HFD exposure (Figure 2B). The acute suppression of inflammation through induction of RIDα/β for 1 week does not impact glucose tolerance (Figure 3B). This argues against a direct impact of inflammation on glucose tolerance. However, longer-term induction of RIDα/β profoundly impairs glucose tolerance and insulin sensitivity (Figure 3C–D). Moreover, hyperinsulinemia and a steatotic liver are observed, concomitant with a widespread whitening of BAT and macrophage infiltration, especially into gWAT in the RID^ad^ mice (Figure 3E–F). Thus, adipocyte inflammation, under either normal or obese conditions, is required to maintain normal insulin sensitivity and glucose homeostasis.

### 3.4 The potent anti-inflammatory action of RIDα/β in macrophages does not affect insulin resistance under obesogenic conditions

**Figure 4:**
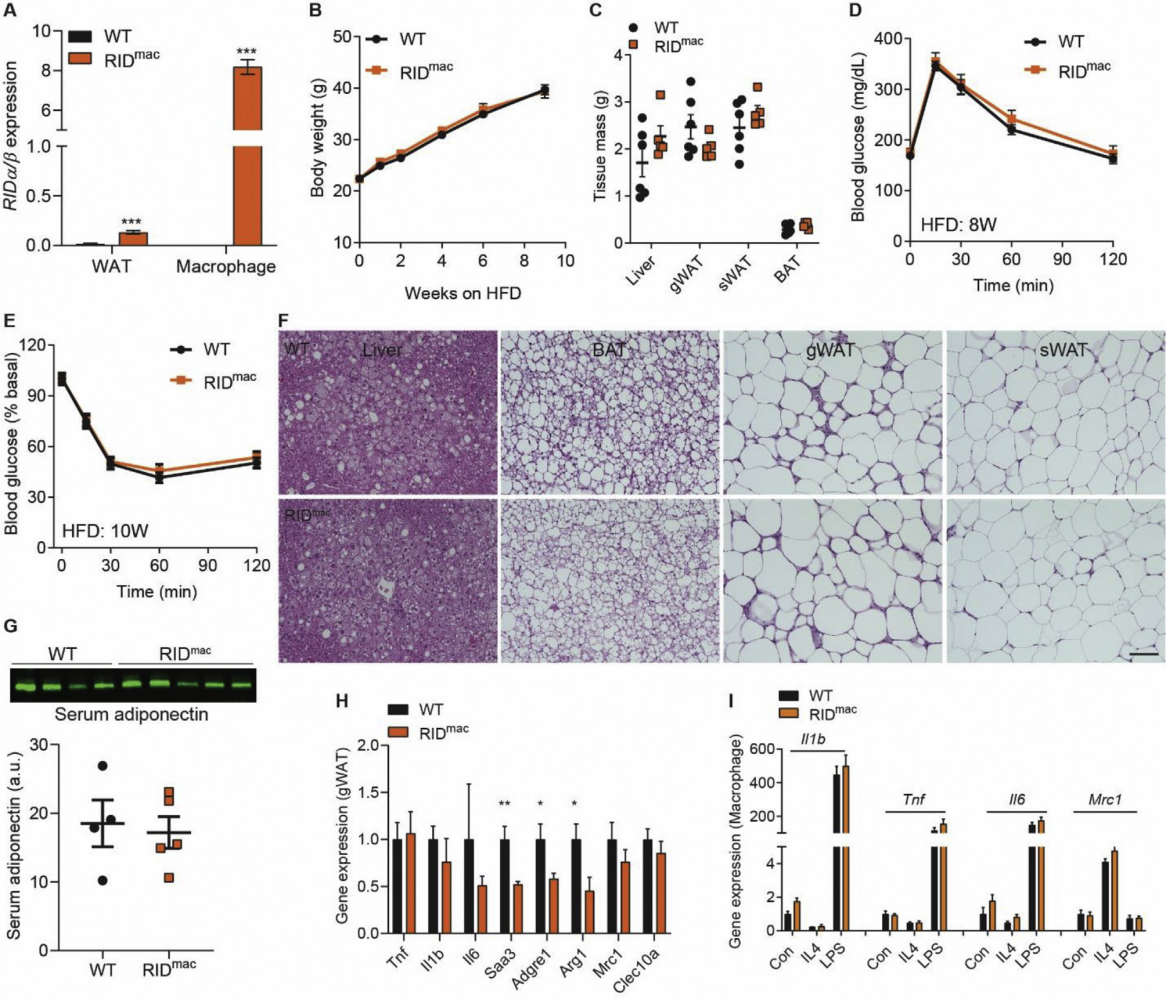
RIDα/β in macrophages do not affect insulin resistance under obesogenic conditions. Mice were fed HFD-dox for 10 weeks. (**A**) Specific induction of *RIDα/β* in macrophages. N = 6/group. (**B**) Similar weigh gain in RID^mac^ mice. N = 15–18. (**C**) Similar liver and fat mass in RID^mac^ mice. N = 5–6/group. (**D-E**) Similar glucose tolerance and insulin tolerance in RID^mac^ mice. N = 15–18/group. (**F**) H&E staining of liver, BAT, gWAT, and sWAT. Scale bar, 100 μm. (**G**) Similar serum adiponectin levels in RID^mac^ mice. N = 4–5/group. (**H**) Inflammatory gene expression in gWAT. N = 10–12/group. (**I**) Inflammatory gene expression in macrophages with IL4 or LPS treatment. N = 3–4/group.

To clarify the inflammatory contributions of macrophages in the progression to insulin resistance, we further took advantage of the modular nature of our RIDα/β mice and crossed them with our Csf1r promoter-driven rtTA mice to generate an inducible macrophage-specific RIDα/β-transgenic mouse model (hereafter referred to as RID^mac^ mice) (Figure 4A). Interestingly, the RID^mac^ mice display similar weight gain, fat mass, and liver mass upon HFD feeding (Figure 4B–C). Moreover, the RID^mac^ mice also display similar glucose disposal and insulin sensitivity (Figure 3D,E). In addition, liver and both BAT and gWAT do not show any histological differences (Figure 3F). Furthermore, the mice have unaltered circulating adiponectin levels (Figure 3G). In RID^mac^-gWAT, some inflammatory genes, including *Adgre1* and *Saa3*, are downregulated; other inflammatory genes such as *Tnf*, *Il1b*, and *Mrc1* are unaltered (Figure 3H). However, these subtle alterations are not sufficient to induce significant changes in adipose tissues. Moreover, macrophages (BMDM) derived from these mice display similar response to IL4 or LPS (Figure 4I), suggesting that RIDα/β does not exhibit a cell autonomous effect on these cells. Thus, the inflammatory response within adipocytes is the main driver for the maintenance of glucose homeostasis, whereas the inflammatory responses of macrophages are not required for the maintenance of metabolic homeostasis in adipose tissue.

### 3.5 Resolving adipocyte inflammation impairs adipose function

**Figure 5:**
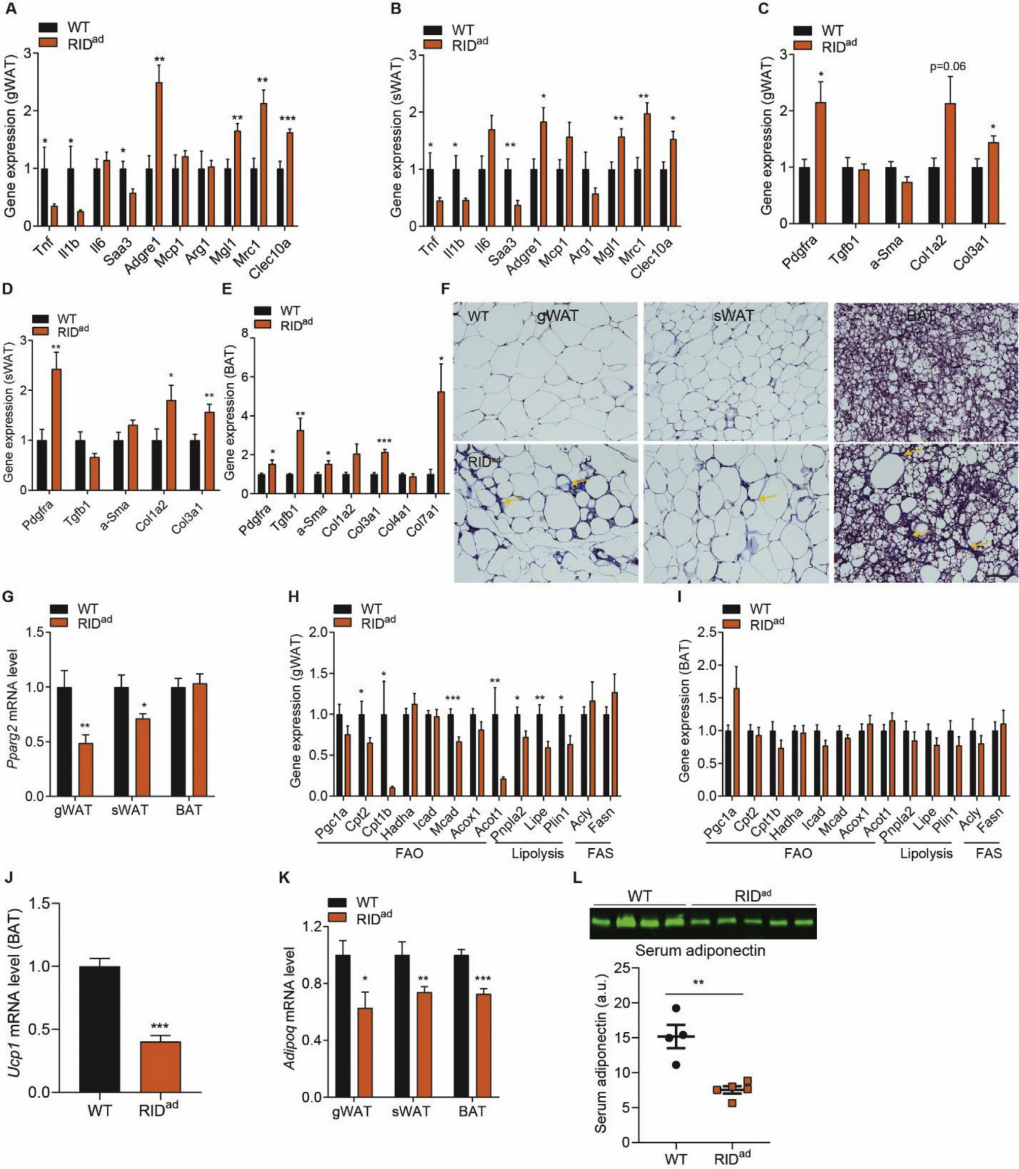
Resolving inflammation impairs adipose tissue function. Mice were fed HFD-dox for 10 weeks. (**A-B**) RIDα/β downregulates M1-like macrophage markers but upregulates M2-like macrophage markers in RID^ad^-gWAT and sWAT. N = 10–14/group. (**C-E**) Upregulated fibrotic markers in RID^ad^-gWAT, sWAT, and BAT. N = 10–14/group. (**F**) Trichrome staining in RID^ad^-gWAT, sWAT, and BAT. Yellow arrows highlight high-level fibrosis in the interstitial space. Scale bar, 100 μm. (**G**) Downregulation of *Pparg2* expression in RID^ad^-gWAT and sWAT but not in BAT. N = 10–12/group. (**H–I**) Expression of genes related to fatty acid oxidation (FAO), lipolysis, and fatty acid synthesis (FAS) in RID^ad^-gWAT and BAT. N = 10–12/group. (**J**) Downregulation of *Ucp1* expression in RID^ad^-BAT. N = 10–14/group. (**K**) Lower adipose *Adipoq* expression in RID^ad^ mice. N = 10–14/group. (**L**) Lower circulating adiponectin levels in RID^ad^ mice. N = 4–5/group.

**Figure 6:**
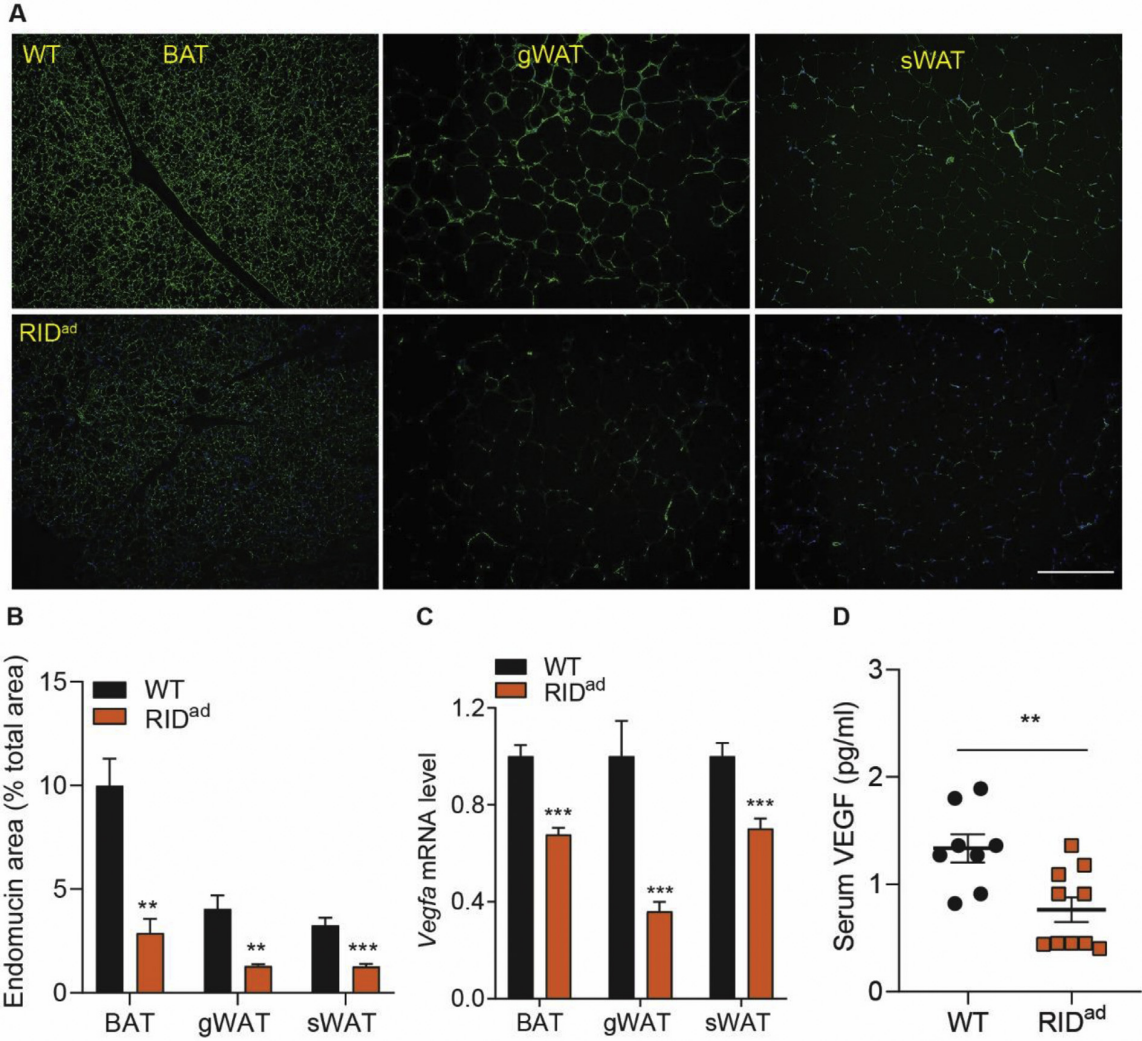
Adipocyte inflammation is required for vascularization. Mice were fed HFD-dox for 10 weeks. (**A-B**) Endomucin staining reveals reduced vascularization in RID^ad^-BAT, gWAT, and sWAT. Scale bar, 300 μm. N = 4/group. (**C**) The angiogenic marker *Vegfa* is downregulated in RID^ad^-BAT, gWAT, and sWAT. N = 8–10/group. (**D**) Circulating VEGF is decreased in RID^ad^ mice. N = 8–10/group.

What are the underlying mechanisms that prompt adipocyte inflammation to lead to insulin resistance? As previously demonstrated, the BAT becomes whitened and gWAT is more infiltrated with macrophages in the RID^ad^ mice, suggesting a multilevel dysfunction of adipose tissues. Indeed, RIDα/β effectively reduces proinflammatory factors locally both in gWAT and sWAT, especially the M1-like macrophage markers that include *Tnf*, *Il1b*, and *Saa3*. However, *Adgre1* is elevated along with a number of M2-like macrophage markers that include *Mgl1*, *Mrc1*, and *Clec10a* (Figure 5A–B). Thus, we could argue that RIDα/β induces macrophage polarization toward a M2-like direction. Moreover, the expression levels of a number of additional fibrogenic genes are upregulated in all fat pads in the RID^ad^ mice (Figure 5C,E), reflecting enhanced fibrosis. This is further supported by trichrome staining, which suggests more collagen production, especially in the interstitial space of RID^ad^-gWAT and BAT (Figure 5F). Moreover, *Pparg2*, the master regulator of adipogenesis, is markedly downregulated in RID^ad^-gWAT and sWAT (Figure 5G), suggesting a lower propensity to adipogenesis. This is consistent in aP2-RID mice [19]. We further assessed the metabolic pathways. In RID^ad^-gWAT, a number of genes related to fatty acid oxidation (FAO) and lipolysis are downregulated, whereas genes related to fatty acid synthesis (FAS) remain unchanged (Figure 5H). However, none of these pathways are altered in RID^ad^-BAT (Figure 5G, I). Nevertheless, *Ucp1* expression is markedly reduced in RID^ad^-BAT (Figure 5J). Thus, adipocyte inflammation has a profound effect on adipose tissue metabolism. Furthermore, *Adipoq* expression is downregulated in all of the fat pads examined, resulting in the lower circulating adiponectin levels in RID^ad^ mice measured in the serum (Figure 5K-L). We further examined adipose tissue vascularization. As determined by the endothelial marker endomucin, vascularization is significantly decreased in all of the fat pads from the RID^ad^ mice (Figure 6A-B). Moreover, *Vegfa*, another critical angiogenesis marker, is significantly reduced, leading to lower circulating levels (Figure 6C-D). Therefore, suppressing adipocyte inflammation leads to widespread adipose tissue dysfunction, prompting us to question the wisdom of anti-inflammatory interventions to improve metabolic health.

## 4 Discussion

Chronic inflammation is a complex process that frequently positively correlates with many metabolic diseases, such as obesity, diabetes, and cardiovascular disease. To dissect the specific inflammatory contributions of adipocytes and macrophages, we blocked inflammatory pathways specifically in these two cell types. We found that adipocyte-derived inflammation, rather than macrophage-derived inflammation, is crucial for maintaining adipose function and systemic insulin sensitivity. These findings highlight the beneficial role that inflammation plays in adipocytes. It also questions the general usefulness of anti-inflammatory interventions intended to improve insulin sensitivity in obesity.

The inducible RID^ad^ mice display similar phenotypes as we previously reported in aP2-RID mice [19] in terms of reduced fat mass, hepatosteatosis, and insulin resistance. Energy expenditure and calorie intake are not altered in aP2-RID mice. However, these mice display prominent issues with intestinal permeability and colitis; treatment with antibiotics improved these negative phenotypic changes, suggesting an important role of adipose tissue as an endotoxin barrier and “sponge” that effectively absorbs endotoxin. Previous studies demonstrated that reducing intestinal permeability profoundly affects glycemic control, inflammation, adipose development, and weight gain [26]. Thus, we speculated that reduced fat mass and weight gain is at least in part due to impairments in the crosstalk between the intestinal tract and adipose tissue. This may not only affect nutrient absorption, but may also worsen adipose development driven by endotoxins from the leaky gut. Further studies will be needed to clarify this mechanism.

Inflammation is a protective response to potentially harmful stimuli, such as challenges from different organisms and tissue damage, with the intent to resolve these challenges by initializing a repair process ultimately aimed to restore tissue structure and function [27]. Adipocytes exhibit the capacity to rapidly remodel in response to fasting-refeeding and chronic HFD challenge [28,29]. Moreover, healthy adipocytes preserve this capacity and retain considerable metabolic flexibility, which is critical for the maintenance of whole body insulin sensitivity [30]. Both lipolysis and inflammation are enhanced upon fasting and reduced upon refeeding in adipose tissue [31,32]. These two processes seem to reciprocally regulate each other in adipocytes. Impairing either pathway disturbs adipocyte function and subsequently negatively impacts insulin sensitivity. In this manner, impairing inflammatory pathways in adipocytes results in postprandial hyperglycemia and insulin resistance, as seen in the chow-fed RID^ad^ mice. Moreover, these metabolic phenotypes further exacerbate upon HFD challenge. Of note, the suppression of adipocyte inflammation after pre-existing obesity has an even further detrimental effect in terms of insulin resistance. Therefore, the positive contributions of inflammation become even more critical in a more stressed microenvironment, as seen for hypertrophic adipocytes in the context of obesity that are more susceptible to inflammatory insults and cell death [33]. Comparable observations are also seen in adipocyte-specific IL6 deficient mice. As a pro-inflammatory cytokine, deletion of IL6 in adipocytes increases adipose macrophage infiltration and promotes obesity-induced insulin resistance [34]. In addition, the manipulation of the downstream inflammatory pathways can also regulate glucose hemostasis, as seen in IKKβ-deficient mice. IKKβ is the primary kinase that promotes inflammation by activating NF-κB in response to proinflammatory factors, such as TNFα and IL1β. Deletion of IKKβ in adipocytes unexpectedly enhances overall adipose tissue inflammation and worsens insulin resistance in HFD-fed mice [35]. Thus, inflammatory pathways in adipocytes are indeed indispensable for maintaining systemic insulin sensitivity.

Importantly, however, we have to differentiate these adipocyte-specific manipulations of inflammatory pathways from systemic elimination in all tissues of various inflammatory components, including members of the NFκB pathway that results in improvements in metabolic homeostasis due to suppression of inflammation in locations beyond adipose tissue. To our surprise, RIDα/β expression in macrophages does not affect HFD-induced insulin resistance. There may be an unknown mechanism in macrophages to overcome the effects of RIDα/β. However, the specific cellular source of a cytokine may be important. In contrast to adipocyte-specific IL6-deficient mice, the deletion of IL6 in both myeloid cells and muscle cells suppresses adipose tissue macrophage infiltration and either improves or at least minimally does not negatively affect insulin resistance [34]. Moreover, the deletion of IKKβ in hepatocytes reduces inflammation and retains liver insulin sensitivity, whereas its deletion in macrophages reduces systemic inflammation and improves global insulin sensitivity [36]. These phenotypes are in sharp contrast to those in the adipocyte-specific IKKβ-deficient mice [35], as previously discussed. Thus, the cell-type specific responses to cytokines may account for the distinct effects on insulin sensitivity. Moreover, the cell–cell communications within a specific tissue context also need to be appreciated. We fail to see a cell autonomous effect of suppressing inflammatory signaling by RIDα/β, as it does not seem to affect adipogenesis and adiponectin production in isolated adipocytes [19], nor does it affect the response to IL4 or LPS stimulation in macrophages in vitro. Thus, we propose that in adipocytes, suppressing inflammatory pathways may impair their response to external stimuli and thereby cause adipocyte dysfunction. This defect can further impact the immunological response and eventually lead to adipose tissue damage and insulin resistance. However, given the complexity of the inflammatory network in adipose tissue, particularly in the context of obesity, the innate and adaptive immune systems certainly also play an important role [37]. Nevertheless, why and how adipocytes exhibit this unique response to inflammation remains unresolved.

We further demonstrate that defects in adipocyte inflammation enhance fibrosis and reduce vascularization in adipose tissues upon obesity. Healthy expansion of adipose tissue requires appropriate ECM remodeling. Disruption of this process causes excess collagen deposition in adipose tissues, leading to fibrosis. Macrophages likely play an important role in the fibrogenic process. In contrast to M1-like macrophages, M2-like macrophages are usually considered metabolically beneficial due to their anti-inflammatory potential. For instance, the anti-inflammatory cytokine IL10 prevents TNFα-induced insulin resistance in adipocytes [38]. However, M2-like macrophages may also contribute to the progression of fibrosis and insulin resistance, probably due to their pro-fibrotic properties. In fact, under a state of insulin resistance, WAT displays more fibrosis and increased M2-like macrophages, which are particularly enriched in fibrotic areas [39,40]. Second, M2-like macrophages inhibit adipogenesis and partial ablation of these macrophages indeed improves systemic insulin sensitivity on the bases of enhanced adipose tissue expansion [41]. Thus, M2-like macrophages may constitute a microenvironment that favors fibrosis but not adipogenesis during the progression of insulin resistance. In our research, we did not observe enhanced apoptosis in any adipose tissues (data not shown). Moreover, the total adipocyte number is tightly controlled by differentiation and cell death upon reaching adulthood, regardless of obesity or fat loss [42,43]. Therefore, our findings further support a model in which an M2-like transition may contribute to lower adipogenesis and fibrosis as indicated in the RID^ad^ mice.

The rapid expansion of adipose tissue requires an enhanced supply of oxygen and nutrients from the blood [44]. Failure to enhance angiogenesis in response to expansion results in local hypoxia and causes cell damage [45]. HIF1α is a master regulator responding to hypoxia. The activation of adipocyte HIF1α results in adipose tissue inflammation, fibrosis, and systemic insulin resistance, whereas its loss of function exhibits opposite phenotypes [[46], [47], [48]]. Hypoxia can activate NF-κB and IκB pathways and enhance the release of proinflammatory cytokines such as TNFα [49]. Moreover, defects in adipocyte inflammation impair adipose tissue expansion [19]. Therefore, adipocyte inflammation may exert important adaptive roles to cope with initial injuries upon hypoxia. In conclusion, we demonstrate a beneficial role of adipocyte inflammation in maintaining adipose tissue function and systemic insulin sensitivity.

## Author contributions

PES conceived the study. QZ and PES designed the experiments. QZ conducted most of the experiments. MK and YZ generated the TRE-RID and Csf1r-rtTA mouse lines. YA, ZZ, SZ, IWA, and CMK conducted some experiments, helped with the mouse breeding, and gave helpful suggestions. QZ wrote the manuscript and PES revised it.
